# A Combination of Peppermint Oil and Caraway Oil for the Treatment of Functional Dyspepsia: A Systematic Review and Meta-Analysis

**DOI:** 10.1155/2019/7654947

**Published:** 2019-11-14

**Authors:** Juanjuan Li, Lin Lv, Jiaqi Zhang, Lin Xu, Enjin Zeng, Zedan Zhang, Fengyun Wang, Xudong Tang

**Affiliations:** ^1^Beijing University of Chinese Medicine, Beijing 100029, China; ^2^Xiyuan Hospital, China Academy of Chinese Medical Sciences, Beijing 100091, China; ^3^China Academy of Chinese Medical Sciences, Beijing 100700, China

## Abstract

A combination of peppermint oil and caraway oil (POCO) with its unique properties has been shown clinical benefits for FD. However, the potent statistical data to confirm its effects are lacking. This meta-analysis thus aimed at evaluating the efficacy and safety of POCO compared with placebo in treating patients with FD. We searched CENTRAL, PubMed, EMBASE (Ovid), Web of Science, Google Scholar, China National Knowledge Infrastructure database, Wanfang, and VIP databases for randomized clinical trials (RCTs) up to June 2019. Dichotomous data were shown as a risk ratio (RR) with 95% confidence intervals (CIs). All data were analyzed by Review Manager 5.2 software. The search identified 382 citations, and 5 RCTs (578 participants) were included. POCO showed a statistically significant effect in global improvement of FD symptoms (RR for not much or very much improvement 0.59, 95% CI: 0.49 to 0.71, *P* < 0.00001, *I*^2^ 36%, NNT 3) and improvement in epigastric pain (RR 1.61, 95% CI: 1.28 to 2.03, *P* < 0.0001, *I*^2^ 0%, NNT 3). There were no significant differences in the total number of adverse events between POCO and placebo (NNH 40). In conclusion, this is the first meta-analysis to assess the effects of POCO in FD. POCO is an effective and safe short-term treatment for FD. However, current findings are based on smaller sample sizes and low/very low quality of the evidence. More well-designed RCTs with large sample sizes of FD patients are required.

## 1. Introduction

Functional dyspepsia (FD) is a common functional gastrointestinal disorder, characterized by one or more of the persistent symptoms: bothersome postprandial fullness, early satiation, epigastric pain, and epigastric burning, in the absence of structural disease to explain these symptoms [1]. The condition has a high population prevalence of 5% to 40% worldwide [2]. It impairs patients' quality of life [3] and work performance and also incurs a substantial economic burden [4, 5]. Effective clinical management of FD is therefore extremely important.

Unfortunately, the pathophysiology of FD remains incompletely understood, although some mechanisms such as gastrointestinal motility disorder, visceral hypersensitivity, and psychological factors have been implicated [6]. Currently, proton-pump inhibitors (PPIs), prokinetics, and psychotropic drugs have been proposed as treatments for FD. However, each gets its own advantages and limitations. The benefits of these approaches are still controversial [7]. In recent years, there has been growing interest in complementary and alternative (CAM) therapies for FD, and clinical trials on herbal preparations increasing over the last few years.

Peppermint oil, an extract of fresh leaves of peppermint, with L-menthol as a major constituent, exerts Ca^2+^ channel blocking properties and contributes to gastrointestinal smooth muscle relaxation [8]. Peppermint oil has been widely used as a spasmolytic agent in the treatment of irritable bowel syndrome (IBS) [9]. For the management of FD, peppermint oil or L-menthol is used in combination with caraway oil. Pharmacodynamic studies have reported that a combination of peppermint oil and caraway oil (POCO) may have a prokinetic effect [10, 11] and interact synergistically in attenuating postinflammatory visceral hyperalgesia [12], all of which might contribute to the therapeutic benefit for FD.

Up to now, several clinical trials have assessed the efficacy of POCO in patients with FD, and its clinical effects appear to be promising [13]. However, there is a lack of potent statistical data to reach a definitive conclusion. So we conducted this meta-analysis to investigate the efficacy and safety of POCO compared with placebo for the treatment of FD.

## 2. Materials and Methods

This systematic review adhered to the Preferred Reporting Items for Systematic Reviews and Meta-Analysis (PRISMA) statement ([Supplementary-material supplementary-material-1]). We have registered the protocol on PROSPERO (CRD42019139647), and the records can be accessed at https://www.crd.york.ac.uk/PROSPERO.

### 2.1. Search Strategy and Study Selection

We performed a preliminary search of the Cochrane Central Register of Controlled Trials (CENTRAL), PubMed, EMBASE (Ovid), Web of Science, Google Scholar, China National Knowledge Infrastructure database, Wanfang database, and VIP database from inception to June 2019. The search strategies were performed by using a combination of subject headings and text words relating to dyspepsia, caraway oil, and peppermint oil (Search [Supplementary-material supplementary-material-1]). There were not any restrictions on language and publication status, and foreign language papers were translated where necessary.

Randomized, placebo-controlled trials evaluating the effect of a combination of peppermint oil and caraway oil in the treatment of adult patients (aged 18 years and over) with FD were eligible for inclusion. The diagnosis of FD was based on either a clinician's opinion or meeting the Rome I, II, III, IV criteria, with a negative upper endoscopy or insignificant findings to explain symptoms. Trials meeting the following criteria were excluded: (1) cohort studies, cross-sectional studies, case reports, letters, reviews, animal experiments, invitro studies, and expert opinions; (2) studies not placebo-controlled; (3) treatment duration less than 2 weeks; and (4) duplicate publications (only the largest publication kept).

Two authors (L-JJ and Z-EJ) independently reviewed studies retrieved by the search strategy and evaluated the title/abstract for eligibility. To avoid the risk of missing eligible trials, the bibliographies of all primary studies and review articles were also checked for additional studies. Once the articles met the criteria, the full text was reviewed for complete analysis. Any disagreement was settled by discussion or consultation with a third author (LV L).

### 2.2. Outcome Assessment

The primary outcome was the global improvement of FD symptoms and improvement in epigastric pain. We used the most stringent definition of overall symptom improvement if more than one definition of symptom improvement was given. The secondary outcome was adverse events (AEs).

### 2.3. Data Extraction

Two authors (L-JJ and Z-EJ) independently extracted clinical data from each included study. When they disagreed, a third author (LV L) resolved the issue. One author (L-JJ) entered it into Review Manager 5.2 (RevMan 2012). Two authors (Z-EJ and LV L) double-checked the accuracy of this process by comparing the study reports with how the data were presented in the systematic review. Extracted data included the following: first author's name, year of publication, study design, setting, country of origin, sample size, diagnostic criteria of FD, dosage regimen in the treatment group, therapy duration, and whether or not IBS excluded. Data were managed and analyzed according to an intention-to-treat analysis, with all dropouts assumed as treatment failures.

### 2.4. Assessment of Risk of Bias

The methodological quality of the included studies was assessed using the Cochrane Collaboration's risk of bias tool [14]. We graded each potential source of bias as high, low, or unclear based on the following domains: random sequence generation, allocation concealment, blinding of participants and personnel, blinding of outcome assessment, incomplete outcome data, selective outcome reporting, and other bias. Disagreements were then resolved by consensus.

### 2.5. Data Synthesis and Statistical Analysis

We analyzed dichotomous data as a risk ratio (RR) with 95% confidence intervals (CIs) and continuous data as a mean difference (MD) or standardized mean difference (SMD) with 95% CI. For dichotomous data, the number needed to treat (NNT) and the number needed to harm (NNH) were calculated using the formula NNT or NNH = 1/(control event rate × (1 − RR)). Both the *I*^2^ statistic and *χ*^2^ test were calculated to assess statistical heterogeneity. *I*^2^ greater than50% or P less than 0.1 suggested significant heterogeneity [15]. A random-effects model was used when there was significant heterogeneity, and a fixed-effects model was used when the heterogeneity was not significant. Possible sources for heterogeneity were evaluated by sensitivity analyses. Data were analyzed using the software Review Manager, version 5.2.

Besides, we used the Grading of Recommendations, Assessment, Development, and Evaluation (GRADE) approach [16] for the assessment of the quality of the evidence with the consensus of two authors (L-JJ and Z-EJ) and developed “[Supplementary-material supplementary-material-1] in Supplementary material” tables by the GRADE profiler software.

## 3. Results

### 3.1. Study Selection and Characteristics

**Figure 1:**
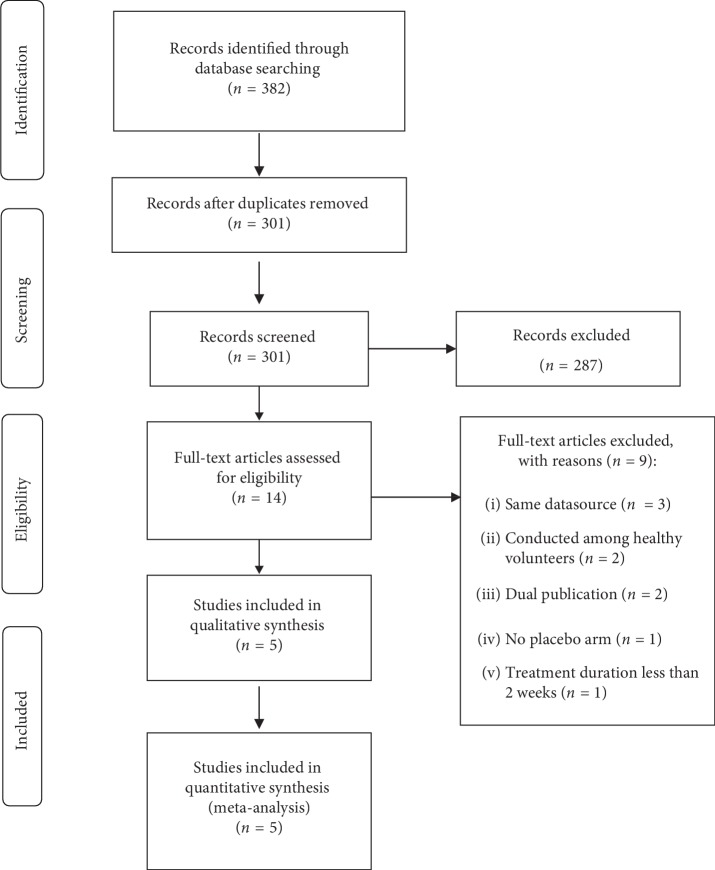
PRISMA flow diagram of the study selection process.

The search strategy identified 382 records, of which 14 papers were targeted for full-article review. A total of five studies involving 578 participants were included in the final meta-analysis. A flow diagram of the study selection process is shown in Figure 1. All five studies were parallel-group multicenter randomized controlled trials (RCTs) with a treatment duration of 4 weeks. The sample size of individual studies varied from 45 to 228. Among the five included studies, three were undertaken in Germany [17–19], one in the USA [20], and one in China [21]. Two studies utilized the Rome III criteria, the other three failed to use any validated criteria, defined by clinical diagnosis and negative investigations. All studies but one included two treatment arms; for that study with three arms (high-dose group, low-dose group, and placebo group) [21], we combined the data from high/low-dose group. It is noteworthy that four studies [17–19, 21] compared the combination of peppermint oil and caraway oil with placebo, another one [20] compared a novel combination of L-menthol (the key active ingredient of peppermint oil) and caraway oil with placebo. Agreement between investigators for assessment of study eligibility was perfect (kappa statistic = 1). The characteristics of the included studies are described in Table 1.

### 3.2. Risk of Bias in Included Studies

All studies mentioned randomization. However, just two articles described the detail of random sequence generation and were rated as low risk of bias for this item. As for allocation concealment, two studies reported adequate methods (i.e., sealed envelopes, stratified by center) and therefore were rated as low risk for this item. It was noteworthy that all five studies were at low risk for performance bias, detection bias, and attrition bias.

**Figure 2:**
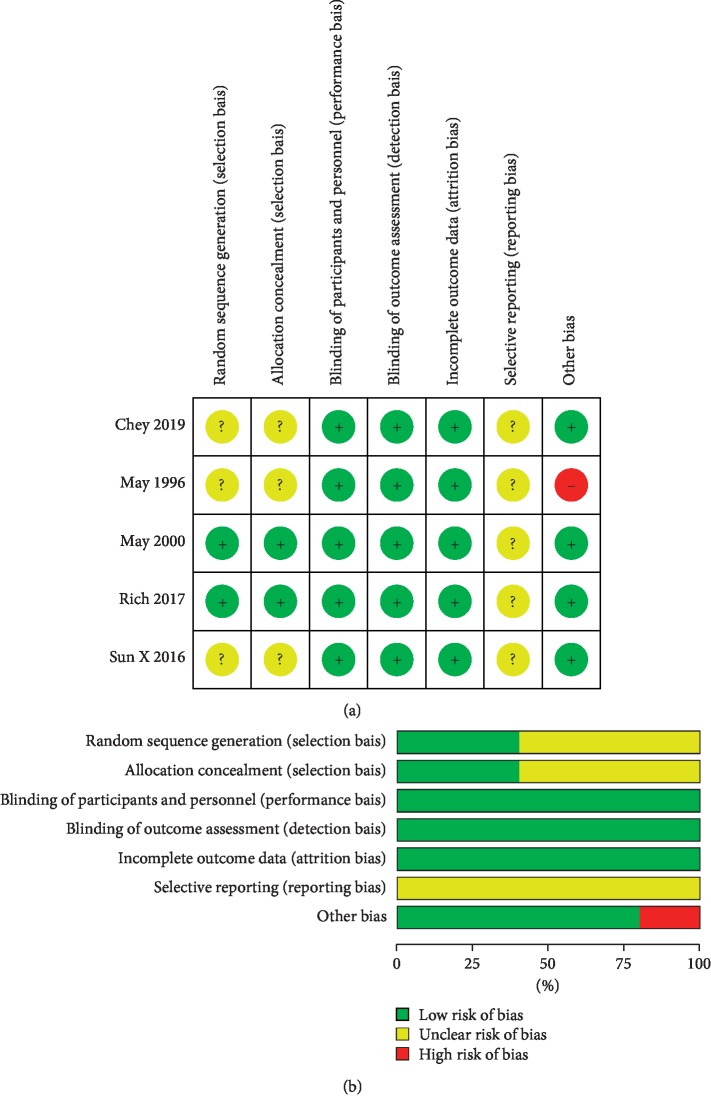
Risk of bias assessment using the Cochrane Collaboration's tool. (a) Risk of bias summary: “+” denotes low risk of bias, “?” denotes unclear risk of bias, and “−” denotes high risk of bias. (b) Risk of bias graph: green, low risk of bias; yellow, unclear risk of bias; red, high risk of bias.

We could not identify protocols or trial registrations to check that studies reported prespecified outcomes (unclear risk of reporting bias). We identified one study in which two groups had a difference in the sex distribution at baseline, with a potential bias on the treatment effect estimate. The risk of bias assessment in the studies is shown in Figure 2.

### 3.3. Global Improvement of FD Symptoms

**Figure 3:**
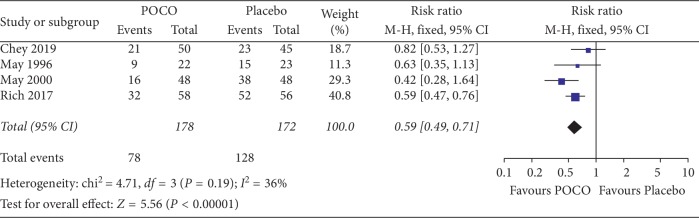
Forest plot comparing a combination of peppermint oil and caraway oil with placebo in patients with functional dyspepsia in terms of not much or very much improvement symptoms.

Four eligible studies with 350 participants reported dichotomous outcomes for the global improvement of FD symptoms [17–20]. These four studies measured much or very much improved symptoms with the clinical global impression (CGI Item 2) investigator rating scale. The patients treated with a combination of peppermint oil and caraway oil (POCO) were statistically significantly more likely to have a global improvement of dyspepsia symptoms compared with participants receiving placebo, with 56.2% (100/178) of the POCO group reporting much or very much improved compared to 25.6% (44/172) of the placebo group (RR 0.59, 95% CI: 0.49 to 0.71, *P* < 0.00001). The NNT was 3 (95% CI: 2 to 5). No statistically significant heterogeneity was detected between studies (*χ*^2^ = 4.71, *df* = 3, *P*=0.19, *I*^2^ = 36%) (Figure 3).

Sun et al. [21] reported global symptoms of dyspepsia as a continuous outcome. The three treatment groups were essentially comparable in total symptom scores at baseline. After 4 weeks of therapy, total symptom scores were significantly lower (mean ± SD) in the POCO group (7.29 ± 5.56, data combined at high- and low-dose groups) than the placebo group (15.42 ± 10.35, *P* < 0.01). The study suggested that POCO would improve global symptoms more significantly compared with placebo.

### 3.4. Improvement in Epigastric Pain

**Figure 4:**
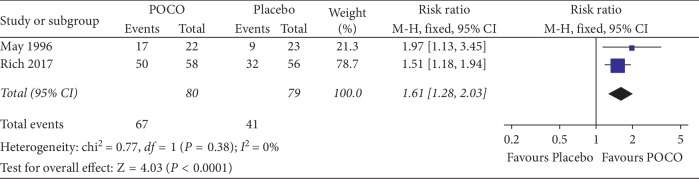
Forest plot showing epigastric pain improvement of a combination of peppermint oil and caraway oil versus placebo.

All five studies reported results of the severity of epigastric pain. Of these 5, two reported improvement in epigastric pain as a dichotomous outcome [17, 19]. Three others [18, 20, 21] reported symptom scores that were not comparable to the other studies. This leaves two studies for meta-analysis. The pooled RR for the effect of POCO (*n* = 80) versus placebo (*n* = 79) on epigastric pain was 1.61 (95% CI: 1.28 to 2.03). There was a statistically significant effect of POCO in improving epigastric pain (*P* < 0.0001), with 82.7% (67/81) of patients receiving POCO showing an improvement compared with 51.9% (41/79) of patients receiving the placebo. The NNT was 3 (95% CI: 2 to 7). No significant heterogeneity was observed (*I*^2^ = 0%, *P*=0.38) (Figure 4).

### 3.5. Adverse Events

**Figure 5:**
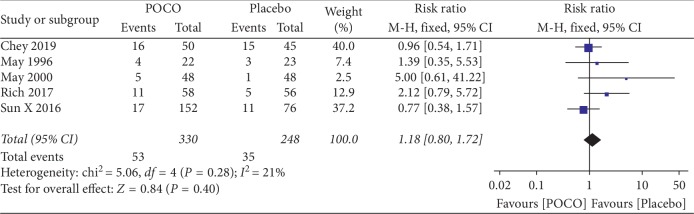
Forest plot of adverse events with a combination of peppermint oil and caraway oil versus placebo in patients with functional dyspepsia.

All five studies reported adverse events (AEs). In total, 53 (16.1%) of 330 patients receiving POCO experienced adverse events compared with 35 (14.1%) of 248 receiving the placebo. No serious AEs were reported. The most common AEs were nausea and eructation. Pooled data in five studies (578 participants) showed no significant difference in reported AEs between POCO and placebo (RR 1.18, 95% CI: 0.80 to 1.72) with no significant heterogeneity between results (*I*^2^ = 21%, *P*=0.28) (Figure 5). The NNH was 40.

### 3.6. Quality of the Evidence

The GRADE system was used to assess the quality of the evidence ([Supplementary-material supplementary-material-1]). In comparing the efficacy of POCO with placebo, the quality of evidence is low in the two outcomes: global improvement of dyspepsia symptoms by the physician and the improvement in epigastric pain. This is due to the risk of bias and serious imprecision. For the outcome “adverse events,” the quality of evidence is very low because of the risk of bias and very serious imprecision (95%CI of pooled data included no effect, very few events).

## 4. Discussions

It is the first attempt to generate RCT data of POCO for the treatment of FD. In this meta-analysis, we evaluated the efficacy and safety of POCO based on five RCTs with 578 patients. The results demonstrated that POCO can significantly improve global symptoms of FD, with an NNT of 3 when data from four studies were pooled. The positive and significant efficacy in terms of improvement in epigastric pain was also shown between the two studies. Moreover, the available data have found that the safety profile of POCO is similar to placebo.

The strength of our findings is that no significant heterogeneity was detected across the studies. Besides, study designs of the included trials were fairly similar and the duration of treatment was identical. We used rigorous methodology as follows and believe that the results reflect the best available current evidence. Firstly, our literature search was comprehensive including all RCTs regardless of publication type and language. Besides, we adopted the intention-to-treat analysis on all data to enhance the robustness of the results.

To the best of our knowledge, a meta-analysis assessing the efficacy and safety of POCO is lacking until now. The current meta-analysis has found that POCO is more effective than placebo on FD symptoms, which may have great clinical significance. POCO's beneficial effects in FD are likely related to its unique prokinetic, anti-inflammatory, gastroprotective, and spasmolytic [8] properties, which can be identified in modern pharmacological studies. In healthy volunteers, this combination has been shown to reduce the frequency and amplitude of contractions in the migrating motor complex (MMC) [11], relax the gall bladder, and slow small intestinal transit [22]. In dyspepsia patients, oral peppermint oil seemed to exert a significant spasmolytic effect in the esophagus, lower stomach, and duodenal bulb [23]. In a rat model, POCO was found to modulate postinflammatory visceral hyperalgesia synergistically. Current studies have underlined impaired duodenal mucosal integrity and low-grade inflammation in the pathogenesis of FD [24, 25]. Both L-menthol and caraway oil also have displayed anti-inflammatory and gastroprotective effects. Oral treatment with L-menthol decreased tumor necrosis factor-*α* (TNF-*α*), interleukin-6 (IL-6) levels, and increased interleukin-10 (IL-10) level in the rat model of gastric ulcers [26]. Besides, L-menthol could relieve inflammatory pain by activating the TRPM8 pathway in vivo [27]. As to caraway oil, it enhanced a significant inhibition of gastric ulcer, which was similar to that induced by omeprazole [28]. It also exerted anti-inflammatory and immunomodulatory effects in TNBS-induced colitis [29]. Thus, the novel combination of caraway oil and L-menthol using microspheres for duodenal release [20] may highlight the clinical benefits partly due to restoring duodenal mucosal integrity. All these findings indicate that POCO has a wide variety of effects on gastrointestinal function, while the precise mechanism of POCO for FD remains unclear. Further studies should be undertaken to fully elucidate it.

There are several limitations to the present meta-analysis, most of which arise from the characteristics of the included studies. First, although we conducted an extensive literature search, only five RCTs were considered in this meta-analysis, and two studies were included in the pooled analysis of improvement in epigastric pain; the number of studies is relatively limited. The funnel plots and Egger's test for publication bias were not feasible. Second, there was substantial variability in the criteria to define FD. Considering some studies failing to use Rome criteria, the efficacy of POCO was not assessed according to the FD subtype. Besides, one of the five trials included patients with comorbid IBS, and the other two did not even screen for IBS. Since POCO and peppermint oil have been confirmed effective in IBS [30, 31], the beneficial effects of POCO in FD likely arise from the inclusion of IBS. Future clinical trials are required to utilize validated Rome criteria, categorize patients as presence or absence of concomitant IBS, and subdivide FD into two subgroups. Finally, all eligible RCTs were based on a short treatment duration of 4 weeks, which means long-term efficacy and safety of POCO in FD are still unknown. Overall, the results of this meta-analysis should be interpreted with caution and confirmed in large-scale, long-term RCTs.

## 5. Conclusions

The evidence suggests that a combination of peppermint oil and caraway oil is an effective and safe short-term treatment for FD. This has significant implications for the management of the condition. However, current findings are based on smaller sample sizes and low/very low quality of the evidence. More large-scale, long-term, and well-designed studies are warranted to resolve these issues.

## Figures and Tables

**Table 1:** Characteristics of the included studies.

Author, published year	Study design	Setting	Country	Sample size	Diagnostic criteria	Dosage regimen in the treatment group	Treatment duration	Were patients with IBS excluded?
Chey et al, 2019 [20]	Double-blind RCT	Multicenter	USA	95	Rome III criteria	COLM-SST, containing 20.75 mg L-menthol (equivalent to 50 mg peppermint oil) and 25 mg caraway oil per capsule, 2 capsules twice daily	4 weeks	Unclear

Rich et al, 2017 [17]	Double-blind RCT	Multicenter	Germany	114	Clinical diagnosis and negative investigations	Menthacarin (a fixed combination of 90 mg peppermint oil and 50 mg caraway oil per capsule), one capsule twice daily	4 weeks	Excluded

Sun et al, 2016 [21]	Double-blind RCT	Multicenter	China	228	Rome III criteria	Enteroplant (a combination of 90 mg peppermint oil and 50 mg caraway oil) per capsule high-dose group: one capsule twice daily, low-dose group: one capsule once daily	4 weeks	Unclear

May et al, 2000 [18]	Double-blind RCT	Multicenter	Germany	96	Clinical diagnosis and negative investigations	PCC/enteroplant (a fixed combination of 90 mg peppermint oil and 50 mg caraway oil) per capsule, one capsule twice daily	4 weeks	Excluded

May et al, 1996 [19]	Double-blind RCT	Multicenter	Germany	45	Clinical diagnosis and negative investigations	Enteroplant (a fixed combination of 90 mg peppermint oil and 50 mg caraway oil) per capsule, one capsule three times daily	4 weeks	Included

RCT, randomized clinical trial; IBS, irritable bowel syndrome.

## Supplementary Materials

Supplementary Materials Table S1: PRISMA checklist. Table S2: summary of findings. The file illustrates the quality of the evidence by the GRADE system. Search strategy S1: the file describes the search strategies performed by using a combination of subject headings and text words relating to dyspepsia, caraway oil, and peppermint oil.
