# Supplementary material for: A Combination of Peppermint Oil and Caraway Oil for the Treatment of Functional Dyspepsia: A Systematic Review and Meta-Analysis
Source: Evid Based Complement Alternat Med. 2019 Nov 14;2019:7654947. doi: 10.1155/2019/7654947 (PMC6885176; doi:10.1155/2019/7654947)
Supplement: Supplementary Materials — Table S1: PRISMA checklist. Table S2: summary of findings. The file illustrates the quality of the evidence by the GRADE system. Search strategy S1: the file describes the search strategies performed by using a combination of subject headings and text words relating to dyspepsia, caraway oil, and peppermint oil. [file 7654947.f1.zip › 7654947.f1/search strategy S1.pdf]

# Search Strategy

**Pubmed search strategy**

1.Search "Dyspepsia"[Mesh] or Dyspepsia* or Indigestion* or FD or NUD

2.Search ("peppermint oil" [Supplementary Concept]) or "Mentha piperita"[Mesh] or peppermint oil or Mentha or piperita or Peppermint or mint oil or oleum menthae or colpermin or Menthol)

3.Search "caraway oil" [Supplementary Concept] or caraway oil or oil of caraway or WS 1520 or Nigella sativa oil

4.Search ( 2 AND 3)

5.Search (Enteroplant or Menthacarin or PCC)

6.6 Search ( 4 OR 5)

7.Search (randomized controlled trial[Publication Type]) OR controlled clinical trial[Publication Type]) OR randomized[Title/Abstract]) OR placebo[Title/Abstract]) OR randomly[Title/Abstract]) OR trial[Title/Abstract]

8.Search( 1 AND 6 AND 7)

**CENTRAL search strategy**

#1 MeSH descriptor: [Dyspepsia] explode all trees

#2 (Dyspepsia or dyspeptic or NUD or FD):ti,ab,kw (Word variations have been searched)

#3 (indigestion* or indigestive):ti,ab,kw (Word variations have been searched)

#4 #1 or #2 or #3

#5 (Peppermint oil or mintoil or colpermin or Menthol or Peppermint):ti,ab,kw (Word variations have been searched)

#6 (caraway oil or oil of caraway or Nigella sativa oil or NS oil or WS 1520):ti,ab,kw (Word variations have been searched)

#7 (Enteroplant or Menthacarin or PCC):ti,ab,kw(Word variations have been searched)

#8 #5 and #6

#9 #7 or #8

#10 #4 and #9

**Embase (OvidSP) search strategy**

1.exp dyspepsia/

2.(Dyspepsia or dyspeptic or NUD or FD).mp.

3.(indigestion or indigestive).mp.

4.1 or 2 or 3

5.exp peppermint oil/

6.exp peppermint/

7.(Peppermint oil or mint oil or oleum menthae or colpermin or Menthol or Peppermint or Enteroplant or Menthacarin or PCC).tw.

8.5 or 6 or 7

9.exp caraway/ or exp carvone/

10.(caraway oil or oil of caraway or Nigella sativa oil or NS oil or WS 1520).tw.

11.9 or 10

12.8 and 11

13.(Enteroplant or Menthacarin or PCC).tw.

14.12 or 13

15.4 and 14

16.random$.tw.

17.placebo.mp.

18.clinical trial.mp.

19.16 or 17 or 18

20.15 and 19

**Web of science search strategy**

#1 TS=(functional dyspepsia or Dyspepsia or dyspeptic or NUD or FD or indigestion or indigestive)

#2 TS=(Peppermint oil or mint oil or oleum menthae or colpermin or Menthol or Peppermint )

#3 TS=(caraway oil or oil of caraway or Nigella sativa oil or NS oil or WS 1520)

#4 TS=(Enteroplant or Menthacarin or PCC)

#5 #3 AND #2

#6 #5 OR #4

#7 TS=(randomized controlled trial or controlled clinical trial or placebo or random* or trial)

#8 #1 and #6 and # 7

**Google scholar search strategy**

("functional dyspepsia" OR "non ulcer dyspepsia" ) and ("Peppermint oil" OR "oleum menthae" OR colpermin OR Menthol)and ( "caraway oil" OR "Nigella sativa oil" OR "oil of caraway") and (randomized controlled trial or placebo)

**China National Knowledge Infrastructure database search strategy**

SU= 'dyspepsia' and SU = ('peppermint oil' + ' peppermint ' + 'caraway' )

**Wan Fang database search strategy**

SU:(functional dyspepsia) * SU: ('peppermint oil' + ' peppermint ' + 'caraway').

**VIP database search strategy**

Title or abstract: (dyspepsia) and title or abstract: ('peppermint oil' + ' peppermint ' + 'caraway').
