# Supplementary material for: A Combination of Peppermint Oil and Caraway Oil for the Treatment of Functional Dyspepsia: A Systematic Review and Meta-Analysis
Source: Evid Based Complement Alternat Med. 2019 Nov 14;2019:7654947. doi: 10.1155/2019/7654947 (PMC6885176; doi:10.1155/2019/7654947)
Supplement: Supplementary Materials — Table S1: PRISMA checklist. Table S2: summary of findings. The file illustrates the quality of the evidence by the GRADE system. Search strategy S1: the file describes the search strategies performed by using a combination of subject headings and text words relating to dyspepsia, caraway oil, and peppermint oil. [file 7654947.f1.zip › 7654947.f1/Table S2_Summary of Findings.pdf]

**a combination of peppermint oil and caraway oil compared to placebo for functional dyspepsia**

**Patient or population:** patients with functional dyspepsia
**Settings:** Outpatients
**Intervention:** a combination of peppermint oil and caraway oil
**Comparison:** placebo

| Outcomes | Illustrative comparative risks* (95% CI) | | Relative effect (95% CI) | No of Participants (studies) | Quality of the evidence (GRADE) | Comments |
|---|---|---|---|---|---|---|
| | Assumed risk | Corresponding risk | | | | |
| | **Placebo** | **a combination of peppermint oil and caraway oil** | | | | |
| **global improvement of FD symptoms** | **744 per 1000** | **439 per 1000** (365 to 528) | **RR 0.59** (0.49 to 0.71) | 350 (4 studies) | ⊕⊕⊝⊝ **low**[1,2] | measurement of not much or very much symptom improvement by the physician. |
| **Improvement in epigastric pain** | **519 per 1000** | **836 per 1000** (664 to 1000) | **RR 1.61** (1.28 to 2.03) | 159 (2 studies) | ⊕⊕⊝⊝ **low**[1,2] | |
| **Adverse events** | **141 per 1000** | **167 per 1000** (113 to 243) | **RR 1.18** (0.8 to 1.72) | 578 (5 studies) | ⊕⊝⊝⊝ **very low**[1,3] | |

*The basis for the **assumed risk** (e.g. the median control group risk across studies) is provided in footnotes. The **corresponding risk** (and its 95% confidence interval) is based on the assumed risk in the comparison group and the **relative effect** of the intervention (and its 95% CI).

**CI:** Confidence interval; **RR:** Risk ratio;

GRADE Working Group grades of evidence
**High quality:** Further research is very unlikely to change our confidence in the estimate of effect.
**Moderate quality:** Further research is likely to have an important impact on our confidence in the estimate of effect and may change the estimate.
**Low quality:** Further research is very likely to have an important impact on our confidence in the estimate of effect and is likely to change the estimate.
**Very low quality:** We are very uncertain about the estimate.

[1] downgraded one level due to study limitations: one study were considered to be high risk of bias
[2] Downgraded one levels due to serious imprecision: very few events
[3] Downgraded two levels due to very serious imprecision: 95% CI of pooled data included no effect and very few events.
